# Adapting the Patient Priorities Care Framework: Innovations in Technology, Surgery, Culture, and Clinical Care

**DOI:** 10.1093/geroni/igaf122.2021

**Published:** 2025-12-31

**Authors:** Melissa Hladek, Mary Tinetti

## Abstract

The Patient Priorities Care (PPC) framework aligns healthcare decisions with the values, goals, and care preferences of older adults. In geriatric primary care, multiple studies have shown that it is feasible, reduces treatment burden, improves collaborative communication and improves health outcomes. As a result, there is increased interest in applying this framework to other communities and situations. This symposium presents four interrelated adaptations of PPC—technological, surgical, cultural, and clinical—that extend its applicability beyond traditional chronic disease management in primary care to address decision-making, digital inclusion, and underrepresented populations. The VISTA (Values Informed Solutions for Technology Adoption) study applies PPC principles in the digital space, helping older adults learn technology to support their goals, thus broadening PPC’s reach beyond clinical settings. Similarly, in surgical decision-making, the Patient Priorities-Aligned Intervention Decisions (PPAID) tool provides structured guidance to ensure that procedures align with patient-defined outcome goals, offering a model for value-driven intervention choices. Recognizing the role of cultural context, a web-based adaptation of the “My Health Priorities” program is being tailored to older African Americans with multiple chronic conditions, addressing usability and accessibility concerns to ensure equitable patient engagement. Lastly, a PPC-focused geriatric consultative model is being implemented to improve care alignment for older, rural-living veterans with multiple chronic conditions. These adaptations demonstrate the flexibility and scalability of the PPC framework, reinforcing its role in fostering patient-centered decision-making across diverse healthcare settings.

